# Oxygen saturation and perceived discomfort with face mask types, in the era of COVID-19: a hospital-based cross-sectional study

**DOI:** 10.11604/pamj.2021.39.203.28266

**Published:** 2021-07-16

**Authors:** Arinze Duke George Nwosu, Edmund Ndudi Ossai, Okechukwu Onwuasoigwe, Francis Ahaotu

**Affiliations:** 1 Department of Anaesthesia, National Orthopaedic Hospital, Enugu, Nigeria,; 2 Department of Community Medicine, College of Health Sciences, Ebonyi State University, Abakaliki, Nigeria,; 3 Department of Orthopaedics, University of Nigeria Teaching Hospital, Ituku-Ozalla, Enugu, Nigeria,; 4 Department of Orthopaedics, National Orthopaedic Hospital, Enugu, Nigeria

**Keywords:** Face-mask, oxygen saturation, discomfort, COVID-19, healthcare workers

## Abstract

**Introduction:**

the COVID-19 pandemic has necessitated the prolonged use of facemasks by healthcare workers. Facemask non-compliance has been largely blamed on discomfort associated with the mask, and apprehension regarding potential health hazards such as asphyxia from mask usage. We sought to evaluate the impact of different respiratory mask types on the comfort of healthcare workers and their arterial oxygen saturation during periods of active clinical duty.

**Methods:**

we conducted a cross-sectional study on healthcare workers donning different types of facemasks in the normal course of duty. Objective non-invasive determination of arterial oxygen saturation of each participant was done using a portable pulse oximeter. Subjective self-assessment of global discomfort was scored by means of a 11-point numerical scale from 0 (no discomfort) to 10 (worst discomfort imaginable). The user's perceived elements of the discomfort were also evaluated. A statistical significance was accepted when P <0.05.

**Results:**

seventy-six healthcare workers completed the study, and wore the masks for periods ranging from 68-480 minutes. The discomfort experienced with the use of the N95 mask; 4.3 (2.0) was greater than the surgical mask; 2.7 (1.8); P=0.001. No significant change in arterial oxygen saturation was observed with the use of either of the mask types. The tight strapping of the N95 mask was perceived as a contributor to the discomfort experienced with mask usage; P=0.009.

**Conclusion:**

the N95 masks imposed greater discomfort than the surgical masks, but neither of the masks impacted on the arterial oxygen saturation of the healthcare workers.

## Introduction

Facemasks are critical components of personal protective equipment (PPE) for healthcare workers, for reducing employee exposure to respiratory infections. Healthcare workers (HCWs) are at increased risk of respiratory infections, and frontline HCWs could have a 12-fold risk of COVID-19 infection compared with the general community [1]. The recent outbreak of COVID-19 has led to guidelines recommending the routine use of facemasks by the populace, and healthcare workers in particular [2]. Compliance rate and practice of facemask use has remained poor among HCWs in parts of the world [3-5]. Discomfort with facemasks can be considerable and has been a major hindrance to compliance, especially among HCWs who wear them for prolonged periods of time [6,7]. And with this, the fear of untoward physiologic consequences. However, little attention has been paid to these factors that hinder compliance with the recommended PPE protocol, even when they are available. The surgical and N95 masks are the two most widely used types of medical facemasks. The surgical masks are comparatively loose-fitting and have variable filtration efficiency, while the N95 mask owes its acclaimed efficacy over the surgical mask to greater filtration efficiency and lesser leakage around the edges.

The discomfort associated with wearing facemasks may arise from a combination of increased resistance to breathing imposed by the mask filter, increase in temperature, humidity and CO_2_ in the dead space of the mask or pressure from the strapping. Few studies have investigated the physiologic impact of medical masks on human subjects, but the outcomes have been contradictory. Some of these suggest that despite the perceived discomfort with their use, facemasks do not impose clinically relevant adverse effects, even with use over long duration and in hot humid environments [8,9]. But Tong *et al*. found that among pregnant women using N95 mask; while the oxygen saturation was unchanged, a decrease in expired oxygen concentration and increase in expired carbon dioxide concentration occurred with its use, both at rest and during low intensity work [10]. Similarly, Fikenzer *et al*. investigated cardiopulmonary exercise capacity of healthy volunteers wearing the surgical mask and N95 mask and found that pulmonary function parameters, power and comfort level were significantly lower with masks, being worse in the latter [11]. This study aimed to measure the impact of wearing the N95 respirators and the surgical masks, over varying periods of time, on comfort level and arterial oxygen saturation (SpO_2_) of HCWs.

## Methods

**Study design and setting:** this is a cross-sectional, institution-based study conducted in the operating suites of a regional trauma centre in Eastern Nigeria.

**Study population:** all perioperative HCWs engaged in clinical duties in the operating suites of the healthcare facility during the period of June 22, 2020 - July 6, 2020 were eligible for inclusion in the study. However, any HCW having an acute upper respiratory tract infection or symptomatic rhinosinusitis with stuffy nasal passages on the days of the study was excluded. Non-consenting HCWs and those who donned N95 mask with expiratory valve were not enrolled for the study. Participants who had cause to breach the seal of the mask in order to sip a drink or other sundries while participating in the study were also excluded.

**Study definitions:** the subjects consisted of anaesthetists, surgeons, perioperative nurses, anaesthetic nurses and operating department assistants (ODAs), assessed in the normal course of their clinical duty in the operating suites. The sociodemographic data collected from each participant were age, and gender. The clinical data collected were; type of mask, duration of mask usage, level of mask discomfort, perceived elements of mask discomfort, and SpO_2_ (before and after mask usage). Each participant was assessed with the mask type available for their use during the period of the study. Four types of N95 masks were in use; model 1870 N95 respirator (3M, St Paul, MN, USA), model 1860 health care particulate respirator and surgical mask (3M, St Paul, MN, USA), model 2130C N95 Particulate Respirator (Cupped) (Louis M. Gerson Co. Inc. Middleboro, MA, USA), model 1730 N95 Particulate Respirator (Louis M. Gerson Co. Inc. Middleboro, MA, USA). Neither qualitative nor quantitative fit-testing was performed prior to donning the masks. Positive pressure and negative pressure user seal checks were implemented by the wearers of the mask (under supervision); to confirm the seal of the N95 mask to the face. Each participant was counseled to ensure that there was no breach of the mask seal until after the final observation.

**Data collection:** a hand-held Smartsigns® MiniPulse MP 1R (Huntleigh Healthcare Ltd, UK) pulse oximeter with the probe applied to the index finger was used for non-invasive determination of arterial oxygen saturation. The SpO_2_ check was done twice for each participant; the first before donning on the facemask, then repeated before undonning of the mask at any time the participant completes their surgical session. The time for donning and undonning of the mask was recorded for each participant and the interval noted, and recorded as the duration of masking. Each participant was also interviewed for level of discomfort before the mask was removed. The application of discomfort was explained to be global, encompassing not only difficulty with breathing but all forms of uncomfortable feeling which may be related to the mask itself, or the strapping. The subjects´ global discomfort was self-assessed and scored by means of 11-point numerical scale from 0 (no discomfort) to 10 (worst discomfort imaginable). The subjects were also requested to indicate the subjective sensations that contributed to their discomfort from four (4) provided options; they could indicate multiple elements. The options were (i) breathing difficulty (ii) facial irritation/hotness (iii) tight strapping, and (iv) communication difficulty with team members. Ambient conditions in operating suites were adjudged comfortable as air-conditioning was provided, but there was no facility to objectively measure the temperature and humidity conditions; both being factors that may significantly influence discomfort associated with respirator masks.

**Statistical analysis:** data entry and analysis were done using IBM Statistical Package for Social Sciences (SPSS) statistical package version 25. Categorical variables were summarized using frequencies and proportions while continuous variables were summarized using mean and standard deviation. Chi square test was used to compare the difference in proportions of two categorical variables. Student t test was used to compare the difference in mean of two samples while correlation analysis was used to determine the degree of straight-line relationship between two quantitative variables. The level of statistical significance was determined by a p-value of <0.05.

**Ethical considerations:** permission for the study was granted by the Research Ethics Committee of National Orthopaedic Hospital, Enugu, Nigeria [S.313/IV. Protocol number; 1051]. Verbal informed consent was sought and obtained from all the participating subjects. The study was conducted in conformity with the Helsinki declaration regarding ethical principles for research involving human subjects.

## Results

**Figure 1:**
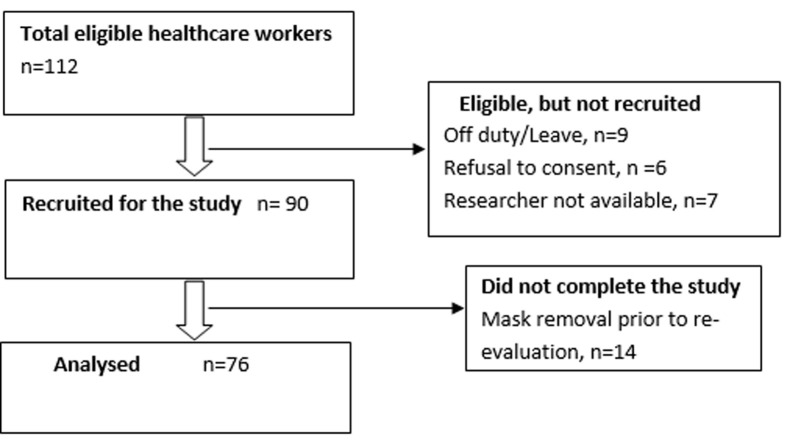
STROBE flow diagram for the study participants

**General characteristics:** ninety perioperative HCWs out of 112 (80%) participated in the study, but only seventy-six HCWs (68%) completed the study after wearing the masks for periods ranging from 68-480 minutes (Figure 1). Thirty-three males and forty-three females participated in the study (Table 1). The age and gender composition of the participants that wore the surgical masks and the N95 masks were similar (Table 1).

**Table 1:** participants' characteristics

Variable	Surgical mask (n=48)	N95 mask (n=28)	p-value
**Age of respondents**			
Mean (SD)	37.9 (8.7)	40.4 (10.1)	0.248
**Age of respondents in groups**			
<35 years	19 (38.6)	9 (32.1)	0.653
35-44 years	18 (37.5)	10 (35.7)	
≥45 years	11 (22.9)	9 (32.1)	
**Gender**			
Male	18 (37.5)	15 (53.6)	0.173
Female	30 (62.5)	13 (46.4)	

**Perceived discomfort from wearing face mask:** the perceived discomfort experienced with the use of the N95 mask, 4.3 (2.0) was significantly greater than the surgical mask, 2.7 (1.8); p=0.001 (Table 2). There was no significant correlation between level of discomfort experienced with either of the mask types, and duration of mask usage (Table 3).

**Table 2:** comparison of outcome variables of the surgical mask and N95 mask

Variable	Surgical mask (n=48)	N95 mask (n=28)	p-value
**Duration of masking (minutes)**			
Mean (SD)	203.0 (86.4)	210.3 (97.1)	0.738
**Perceived level of discomfort**			
Mean (SD)	2.7 (1.8)	4.3 (2.0)	0.001
**Pre-test SpO_2_**			
Mean (SD)	98.1 (0.7)	97.9 (0.8)	0.388
**Post-test SpO_2_**			
Mean (SD)	98.1 (0.8)	97.8 (0.8)	0.114

**Table 3:** correlation of level of discomfort with duration of masking

Variable	Sample size (n)	Pearson correlation (r)	p-value
Surgical mask: correlation of level of discomfort with mask duration	n=48	0.073	0.623
N95 mask: correlation of level of discomfort with mask duration	n=28	-0.225	0.250
N95 and surgical mask correlation of level of discomfort with mask duration	n=76	-0.035	0.765

**Perceived contributors to mask discomfort:** tight strapping of the N95 mask was strongly perceived as a contributor to the discomfort experienced with mask usage; p=0.009 (Table 4). The other perceived elements of discomfort; difficulty with breathing, difficulty in communication with team members and facial irritation, did not produce significant impact on the discomfort associated with facemask use.

**Table 4:** perceived contributors to mask discomfort

Variable	Surgical mask (n=48) N (%)	N95 mask (n=28) N (%)	p-value
**Difficulty in breathing**			
Yes	16 (33.3)	12 (42.9)	0.406
No	32 (66.7)	16 (57.1)	
**Facial irritation/hotness**			
Yes	19 (39.6)	14 (50.0)	0.377
No	29 (60.4)	14 (50.0)	
**Tight strapping**			
Yes	13 (27.1)	16 (57.1)	0.009
No	35 (72.9)	12 (42.9)	
**Difficulty in communication**			
Yes	23 (47.9)	13 (46.4)	0.900
No	25 (52.1)	15 (53.6)	

**Oxygen saturation:** the baseline SpO_2_ (pre-test SpO_2_) in the two groups of participants was similar (Table 2). No significant difference in SpO_2_ was observed after the use of either of the mask types (post-test SpO_2_) (Table 2). The SpO_2_ was unchanged in the majority of the HCWs, with a few recording only slight elevation or reduction with either mask type (Table 5).

**Table 5:** observed pattern of oxygen saturation changes with the surgical mask and N95 mask

Mask type	SpO_2_ reduced N (%)	SpO_2_ unchanged N (%)	SpO_2_ increased N (%)
Surgical (n=48)	11 (22.9)	26 (54.2)	11 (22.9)
N95 mask (n=28)	2 (7.1)	19 (67.9)	7 (25.0)

## Discussion

We did not observe any significant effect on SpO_2_ associated with the use of either mask by the HCWs. However, the HCWs perceived much greater level of discomfort with the N95 mask compared to the surgical mask. There was no correlation between the discomfort perceived while using either mask type, and the duration of their usage. The tight strapping of the N95 mask was perceived as a strong contributor to mask-related discomfort. Tabansi and Onubogu had monitored Nigerian HCWs in different departments over a period of 8 hours in a cross-sectional study, and noted no significant differences in the mean SpO_2_ over the duration, irrespective of the mask types they wore (N95, surgical mask, cloth mask) [12]. Furthermore, the work of Roberge *et al*. which used a treadmill-simulated work schedule to study the physiologic impact of N95 mask on HCWs had also reported no difference in their SpO_2_ from those who wore no mask [13]; lending further support to our finding. In the contrary, one evaluation conducted inside the operating suite among a group of surgeons reported reduction in SpO_2_ among the surgeons who wore surgical masks during surgery [14]. It is pertinent to note that in the said study the group of surgeons who did not wear masks during surgeries lasting less than 30 minutes also experienced significant reduction in SpO_2_ after the procedure; p=0.0006. Based on this observation the authors had postulated that the reduction in SpO_2_ could be attributable to the stress of duty rather than the mask factor. The higher level of discomfort perceived with the N95 use among our HCWs is in tandem with the findings of Scarano *et al*. [15] and Li *et al*. [16]; among Italian HCWs and Hong Kong threadmill exercise volunteers respectively. Similarly, in their evaluation of the effects of donning different mask types by volunteers undergoing simulation exercise, Fikenzer *et al*. reported that whereas masks were generally perceived as uncomfortable, the N95 masks were significantly more uncomfortable than surgical masks [11].

Our evaluation of the perceived contributors of mask discomfort indicated that the HCWs regarded the tight strapping of the N95 mask more than the other subjective elements of discomfort such as breathing difficulty, facial irritation/hotness, and communication difficulty. Another evaluation of the effects of prolonged use of facemask among Indian HCWs also acknowledged the role of tight strapping, but appeared to rank it behind symptoms such as 'excessive sweating around the mouth' and 'breathing difficulty on exertion' [17]. However, while these other symptoms were more prevalent in their cohort than the ear pain from tight strapping, their mere presence may not necessarily infer discomfort. Moreover, some of the symptoms they experienced while wearing the mask may not be mask-related. Our study was specific about mask-related discomfort, as some elements that contribute to it such as 'tight strapping' and 'difficulty in communication with team members' may not be properly classified as symptoms. We could not establish a significant correlation between the level of discomfort felt by the HCWs and the duration of mask usage. In the contrary, Shenal *et al*. [7] and Rebmann *et al*. [8] separately reported increasing levels of discomfort in their cohorts following longer duration of respiratory mask usage. Unlike our study, these two studies had the participants wear the masks for extended periods of 12 hours and 8 hours respectively, following 'fit-testing'. We had earlier informed that our institution lacked the facility for 'fit-testing' as such could not conduct it in our participants. These variations in test conditions could possibly impact on the discomfort level experienced over time.

With the findings of our study, it is considered useful to determine the physiologic impact of mask use on oxygen saturation, the level of discomfort associated with the various mask types and the factors perceived to contribute to the discomfort. This may engender greater confidence and compliance of the HCWs while using the devices, and provide relevant information to biomedical engineers towards reducing the discomfort impacted by the masks. The necessity of these research outputs has earlier been highlighted by Baig *et al*. [18]. The strategic importance of user friendly, comfortable medical mask has also been emphasized in the Harvard Business Review [19]. These will culminate in greater compliance with medical mask protocols by HCWs, and the general populace, thereby ensuring better infection control and safety.

This study has some limitations attributed mainly to technological deficiencies. The N95 masks were of different brands having been donated by different agencies in aid of the government's collaboration against the COVID-19 pandemic. Consequently, as their respective strapping and designs differ these may impact on their physical properties and associated discomfort. 'Fit- testing' is recommended for safe use of respirators such as the N95, and their reliability in preventing disease transmission depends on their fit to the face [20]. This was not feasible in our resource-constrained environment where the test equipment is lacking. Combination of 'fit-testing' and formal 'user seal checks' ensure quality donning of the N95 mask, as the latter alone may be unreliable for detecting respirator leakage [21]. Thus, in the absence of 'fit-testing', leak-proof respiration with the N95 mask could not have been guaranteed by the 'user seal checks' alone which we implemented, and this could have impacted the oxygen saturation and the discomfort level ascribed to the mask. In effect the discomfort level could have been under-estimated, while the SpO_2_ could have been over-estimated while donning the N95 mask. Nevertheless, there lies strength in our study wherein the impact of the masks on the HCWs was assessed in the normal course of their clinical duty, rather than laboratory simulations.

## Conclusion

The N95 respirator mask imposes significantly more discomfort on the HCWs, than the surgical mask. However, neither mask type was associated with any significant change in oxygen saturation during use in routine clinical duty.

### What is known about this topic

- Facemasks are associated with varying levels of discomfort during use;
- The N95 mask is generally credited with greater discomfort than the surgical mask.

### What this study adds

- Neither the N95 mask nor surgical mask impacts a change in subject’s oxygen saturation during use;
- The tight strapping of the N95 mask was perceived as a significant contributor to discomfort, but not the other subjective elements of discomfort such as breathing difficulty, facial irritation/hotness, and communication difficulty.
